# Subdiaphragmatic abscess due to penetration of a duodenal ulcer successfully treated with endoscopic transgastric drainage: a case report

**DOI:** 10.1186/s13256-021-02970-8

**Published:** 2021-07-26

**Authors:** Tomoya Takami, Hiroshi Takihara, Koji Yasuda, Nozomi Kasyu, Hiroyuki Yoshitake, Hiroshi Shintani, Naoki Kataoka, Tomoyuki Yamaguchi, Shinichiro Makimoto

**Affiliations:** Department of General Surgery, Gastroenterology, Kishiwada Tokushukai Hospital, 4-27-1 Kamoricho, Kishiwada, Osaka 596-0042 Japan

**Keywords:** Subdiaphragmatic abscesses, Endoscopic ultrasound-guided drainage, Duodenal ulcer

## Abstract

**Background:**

Subdiaphragmatic abscesses are sometimes caused by intraabdominal infections. We report a case of endoscopic ultrasound-guided transgastric drainage.

**Case presentation:**

A 75-year-old Asian man was referred to our hospital for treatment for upper gastrointestinal bleeding. On admission, blood tests showed a marked inflammatory response, and abdominal computed tomography showed free air in the abdominal cavity and a left subdiaphragmatic abscess. Therefore, the patient was diagnosed with an intraabdominal abscess associated with a perforated duodenal ulcer. Because he did not have generalized peritonitis, fasting and antibiotic treatment were the first therapies. However, because of the strong pressure on the stomach associated with the abscess and difficulty eating, we performed endoscopic ultrasound-guided transgastric drainage. After treatment, the inflammatory response resolved, and food intake was possible. The patient’s condition remains stable.

**Conclusions:**

Drainage is the basic treatment for subdiaphragmatic abscesses; however, percutaneous drainage is often anatomically difficult, and surgical drainage is common. We suggest that our success with endoscopic ultrasound-guided transgastric drainage in this patient indicates that this approach can be considered in similar cases and that it can be selected as a minimally invasive treatment method.

## Introduction

Causes of subdiaphragmatic abscess include abdominal-surgery-related infections, traumatic abdominal injury, upper gastrointestinal perforation/penetration, and intraabdominal infections, such as hepatic/biliary inflammation [1]. Recently, with progress in endoscopic ultrasonographic (EUS) instruments and techniques, transgastric internal fistula drainage for abdominal abscess has been widely performed.

We report a case of subdiaphragmatic abscess caused by a perforated duodenal ulcer treated with EUS-guided transgastric drainage, with a favorable post-treatment course.

## Case presentation

A 75-year-old Asian man presented to another hospital with an approximately 1-month history of epigastric pain after meals. Upper gastrointestinal endoscopy had been performed to investigate his epigastric pain and revealed a peptic ulcer in the duodenum. In anticipation of bleeding from the ulcer in the future, he was referred to our hospital for endoscopic hemostasis without further endoscope observation. He had no relevant past medical history, family history, or psychosocial history. However, he was taking nonsteroidal antiinflammatory drugs for back pain as well as a proton pump inhibitor.

On physical examination, the patient's temperature was 36.8 °C, his blood pressure 40/82 mmHg, heart rate 114 beats/minute, and respiratory rate 18 breaths/minute. His abdomen was flat and soft, with no tenderness. Intestinal peristalsis was normal, and there were no audible vascular sounds over the abdomen.

**Fig. 1:**
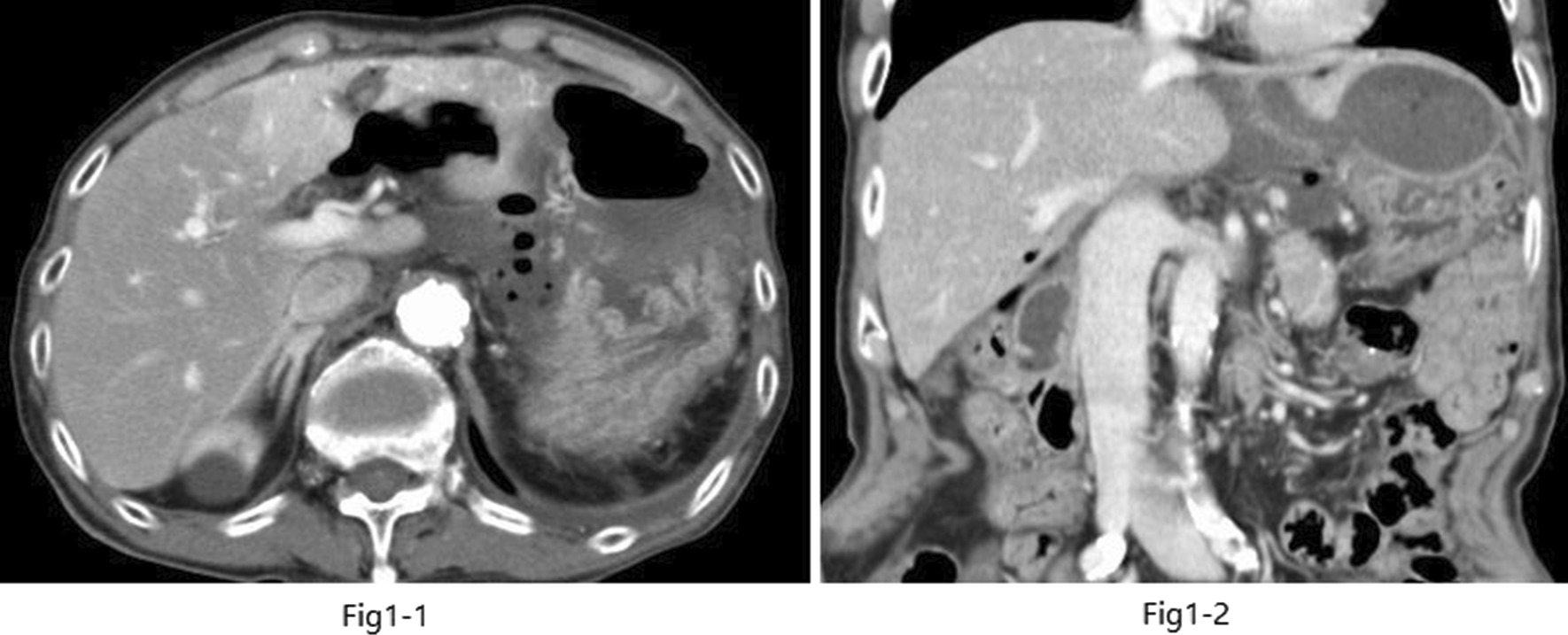
(**1**) Computed tomography scan (coronal view); an abscess is visible pressing on the stomach. (**2**) Computed tomography scan (axial view of the same location as in **1**)

Blood tests revealed a marked inflammatory response, with a white blood cell count of 13,300 cells/μL, C-reactive protein concentration 17.229 mg/dL, hemoglobin concentration 10.1 g/dL, platelet count 304,000 cells/μL, blood urea nitrogen concentration 65.2 mg/dL, and creatinine concentration 1.19 mg/dL. Abdominal computed tomography (CT) showed free air in the abdominal cavity, thickening of the duodenal wall with edema, and an abscess cavity under the left diaphragm (Fig. 1(1, 2)). Abdominal ultrasonography also revealed irregular edematous thickening of the duodenal bulb. Based on the increased inflammatory response, presence of free air in the abdominal cavity, and edema of the duodenal wall, the diagnosis was a perforated duodenal ulcer and intraabdominal abscess.

**Fig. 2:**
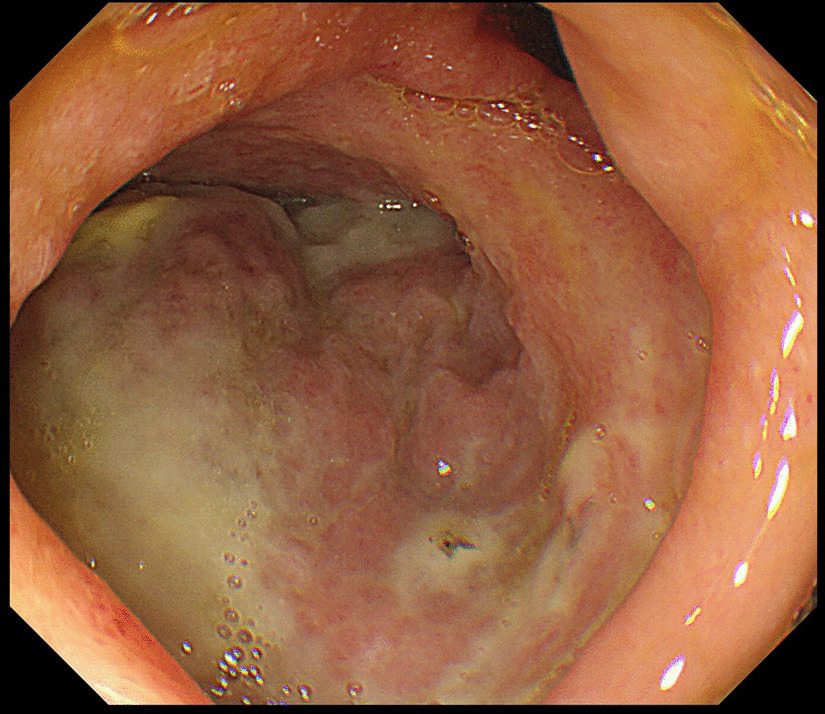
Gastrointestinal fiberscopy. An ulcer is visible as a white mass on the posterior wall of the duodenal bulb

In addition, we considered a diagnosis of localized peritonitis, as a result of the findings, and decided to use conservative treatment. The patient was hospitalized and treated with fasting and 16 days of antibiotic treatment (meropenem 2 g every 12 hours).The clinical course was favorable, and the inflammatory response improved. However, abdominal CT performed 10 days after admission showed that the size of the abscess cavity had changed very little, and that the stomach position was deviated because of pressure from the abscess. Therefore, water intake was not a problem, but food intake was difficult, and we decided that drainage was necessary. CT and echo-guided puncture is usually anatomically difficult in this patient’s abscess location. We considered surgical drainage, but because the abscess was in contact with the stomach, we considered it possible to perform transgastric puncture drainage under EUS guidance, which was also a less invasive procedure. Upper endoscopy performed before EUS revealed an ulcer in the duodenal bulb (Fig. 2). The test for *Helicobacter pylori* was negative. The lesion was ulcerated and staged as A1 according to the Sakita–Miwa classification for gastric ulcers [2]. However, postoperative abdominal CT scans obtained after endoscopy showed no increase in the amount of free air, suggesting that the perforation site was blocked.

**Fig. 3:**
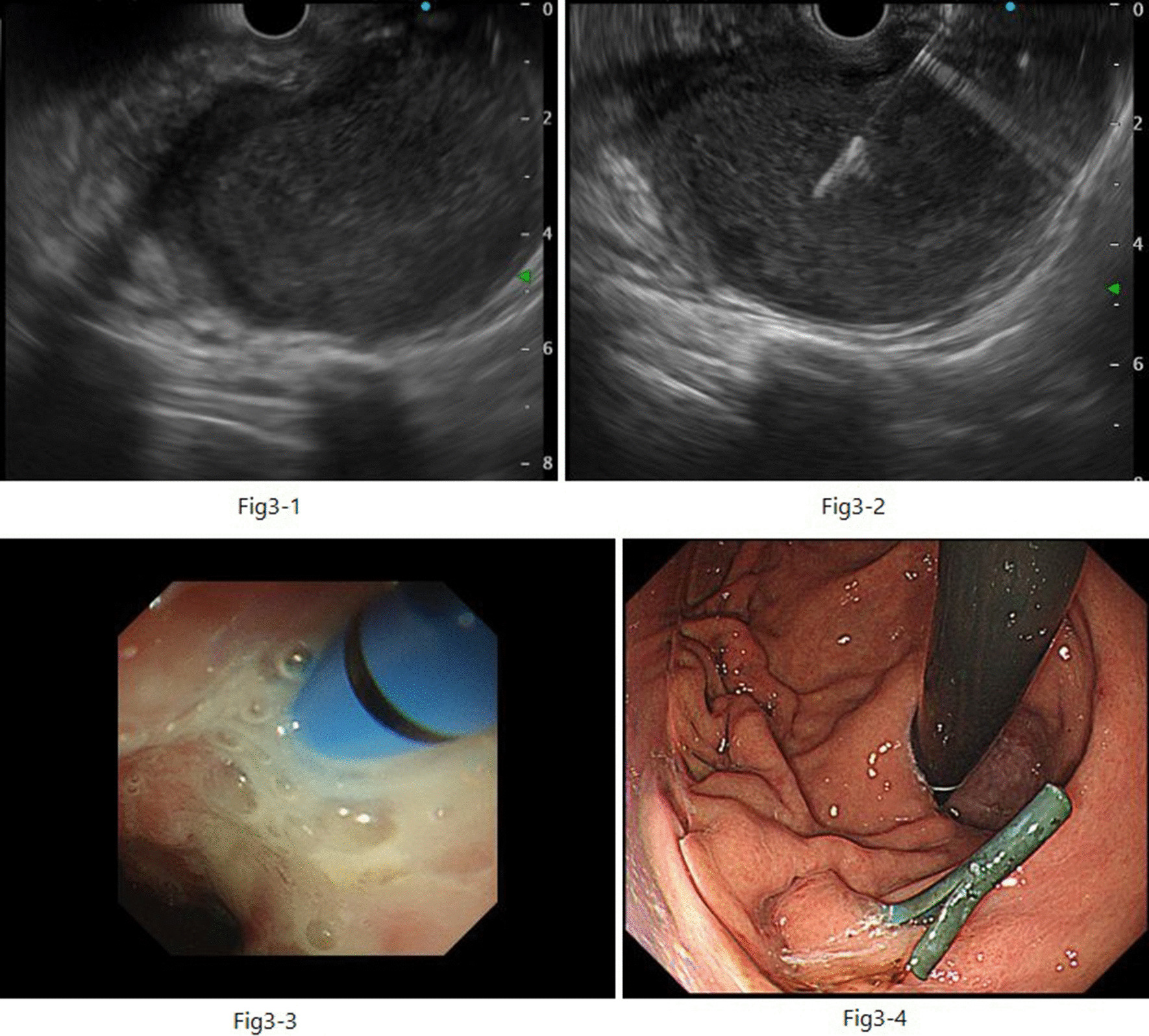
(**1**) Endoscopic ultrasonographic image showing the abscess as a round hypoechoic area. (**2**) Endoscopic ultrasonographic image showing abscess puncture under echo guidance. The white line in the center is the needle. (**3**, **4**) Gastrointestinal fiberscopy (after puncture): white fluid is seen draining from the abscess through the drain

**Fig. 4:**
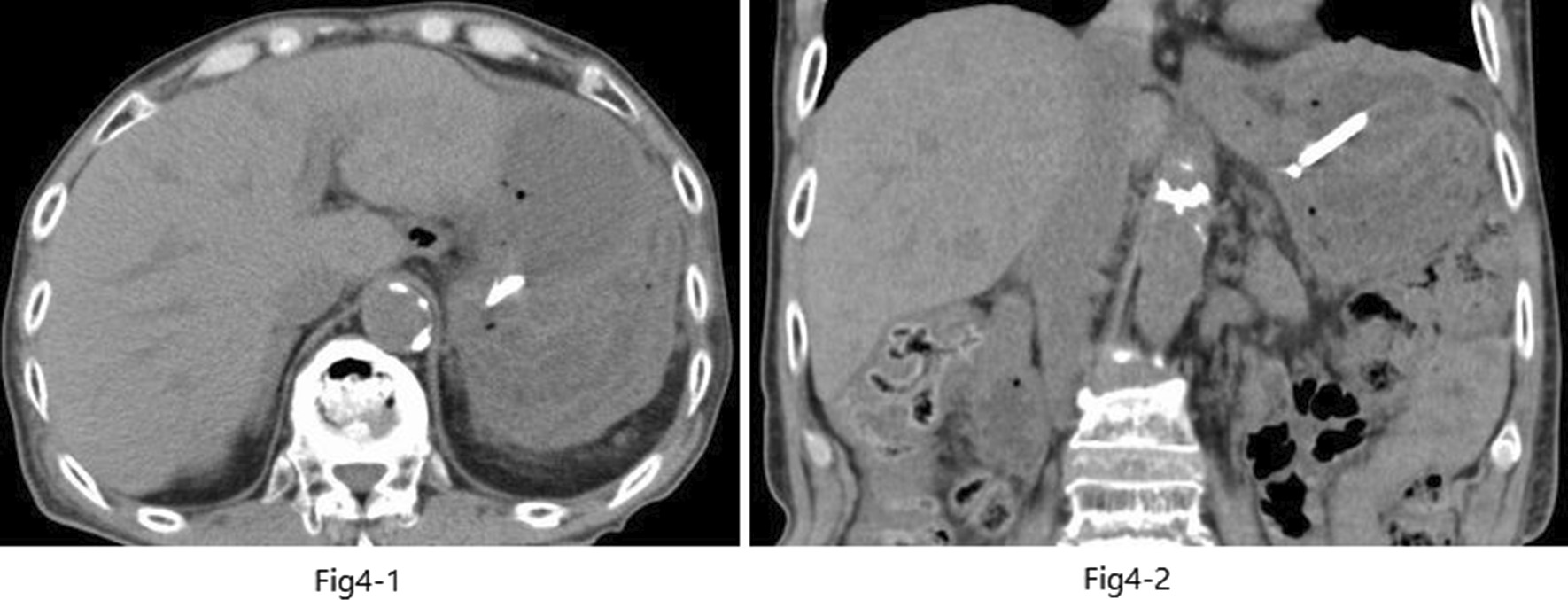
(**1**) Computed tomography scan (coronal view) confirming the drain (white line) placed in the abscess. (**2**) Computed tomography scan (axial view of the same image as in **1**)

**Fig. 5:**
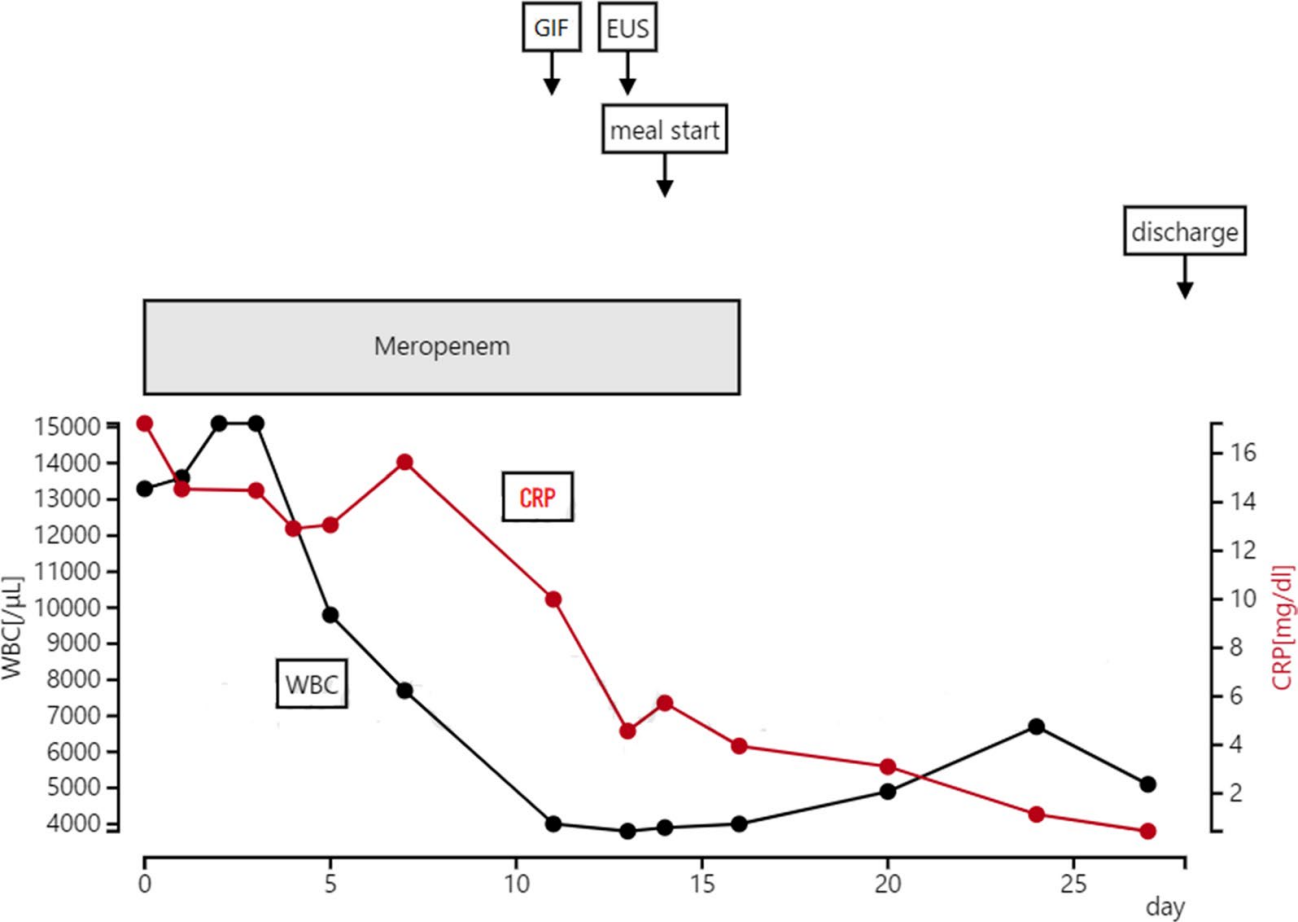
Progress after hospitalization

**Fig. 6:**
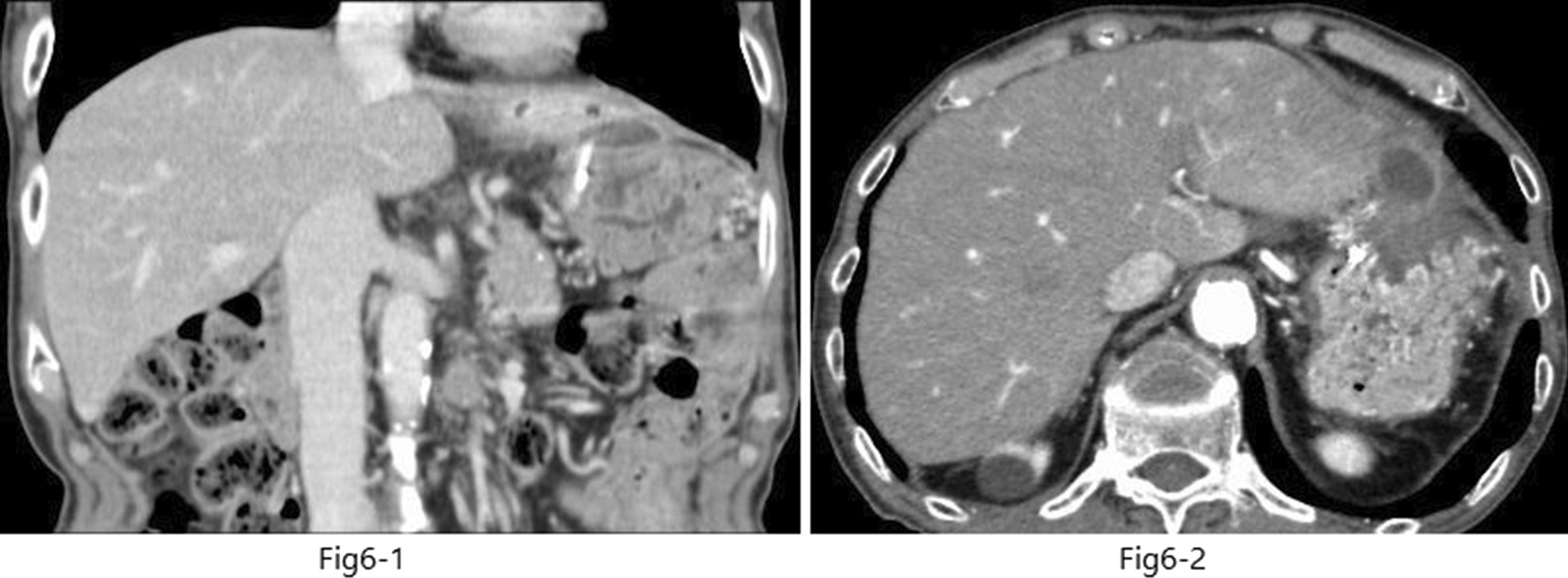
(**1**) Computed tomography scan (coronal view) showing that the abscess is smaller in size. (**2**) Computed tomography scan (axial view of the same image as in **1**)

**Fig. 7:**
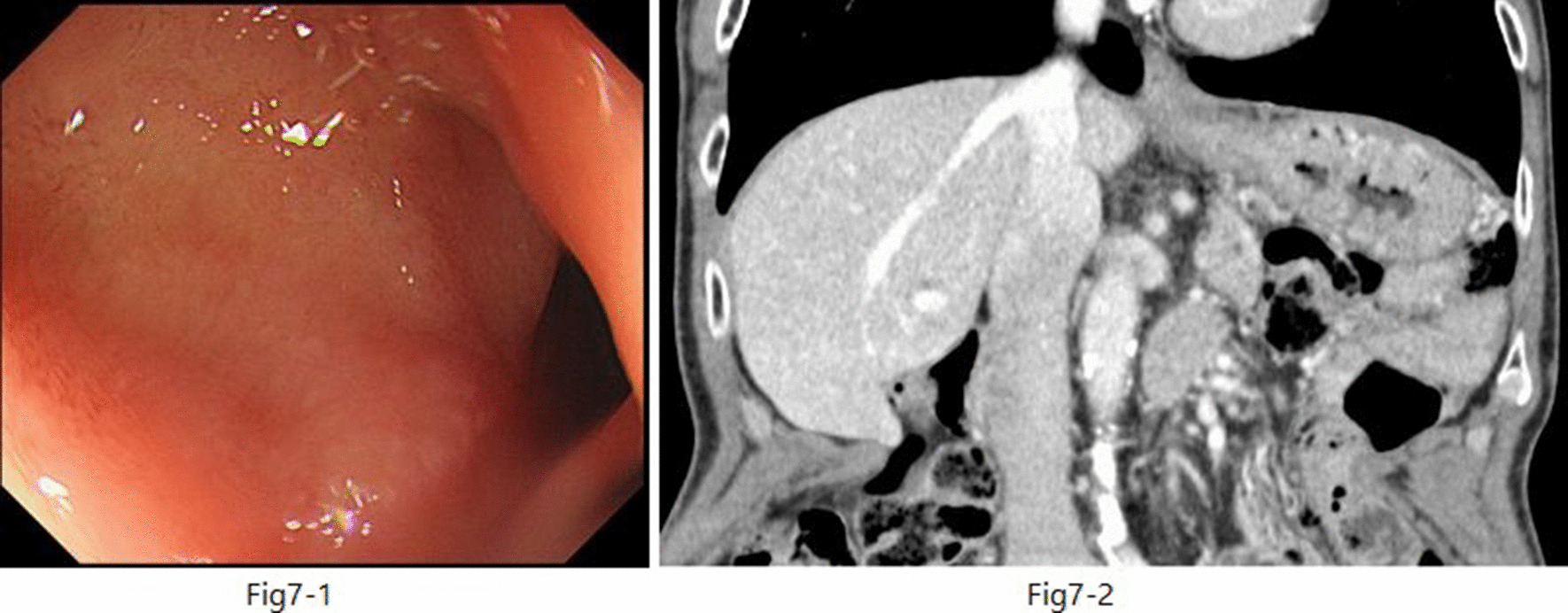
(**1**) Gastrointestinal fiber copy; the ulcer has healed. (**2**) Computed tomography scan (coronal view) showing that the abscess has disappeared and the tube has also been removed

EUS-guided transgastric drainage performed under sedation with midazolam on hospital day 13 confirmed an abscess measuring approximately 4 × 6 cm in the anterior wall of the upper body of the stomach. The abscess was then punctured with a 19-gauge EUS needle (EZ Shot 3; Olympus, Tokyo, Japan). Next, an 8.5-Fr straight plastic stent was placed in the abscess using a 0.025-Fr guidewire (Cheerleader; Pyorax Japan, Yokohama, Japan) (Fig. 3-1–4).The treatment was completed without any particular problems, and the patient’s progress after the treatment was favorable. *Stenotrophomonas maltophilia* was detected in pus from the puncture site but was deemed not to warrant treatment because antibiotic therapy had already been provided. Furthermore, abdominal CT confirmed that the drain was placed in the abscess cavity, and that the abscess cavity diminished over time (Fig. 4(1, 2)). The patient’s course was uneventful after restarting solid food, and the patient was discharged on the 29th hospital day with the drain left in place (Fig. 5). The patient was seen subsequently at our outpatient clinic, but the abscess cavity had shrunk, and the patient’s course was proceeding well (Fig. 6(1, 2)). The drainage tube was removed at a later date when the duodenal ulcer had healed (Fig. 7(1, 2)).

## Discussion

Intraabdominal abscesses often occur following intraabdominal surgery, trauma, severe enteritis, intestinal perforation, and acute pancreatitis [1, 3]. Of these, 30% of episodes are secondary to inflammatory diseases of the abdominal organs due to gastric/duodenal perforation, 20% are related to the liver/biliary system, and 12−31% are related to appendicitis [1, 4]. Most patients with a perforated duodenal ulcer present with peritonitis and require surgery, but in our case, the patient’s general condition was stable, and there were no signs of peritoneal irritation, so conservative treatment was selected. Although the patient improved with conservative treatment, we concluded that drainage was necessary because we predicted that gastric pressure associated with the abscess would be strong, and that food intake would be difficult.

Abscess drainage methods include surgical drainage, percutaneous drainage, and endoscopic drainage. However, subdiaphragmatic abscesses form between the diaphragm and abdominal organs, such as the liver and spleen. As a result, it is difficult to drain these abscesses with a percutaneous approach, and a transthoracic approach is often necessary. However, transthoracic approaches carry the risk of pneumothorax [5]. Therefore, since the abscess cavity was in contact with the stomach in this case, we considered that transgastric drainage was possible under EUS guidance. In addition, we considered endoscopic drainage to be less invasive than surgical drainage and safer to perform than percutaneous drainage.

Since the first report of EUS-guided drainage for a pancreatic pseudocyst [6], the technique has been widely used [7]. The advantages of EUS-guided drainage are: (1) even a deep abdominal abscess can be clearly visualized by ultrasonography; (2) using Doppler mode, puncture can be performed while avoiding blood vessels; (3) under ultrasound guidance, treatment can be performed while watching the real-time video; and (4) there is no risk when it is not desirable to place the drainage tube under the skin because of the risk of self-removal [8]. However, as a disadvantage, the drainage tube is thinner than the tube used for percutaneous drainage, and therefore, there is a possibility of poor drainage when there is food residue in the abscess cavity or when the viscosity is high. Therefore, if EUS-guided drainage is insufficient, it is necessary to consider additional treatment.

Regarding the management after aspiration, it is necessary to consider the risk of infection owing to communication with the digestive tract. Retrograde infection due to increased gastric pressure associated with oral intake is a concern with insufficient drainage in the early stage using an indwelling drain; however, restarting food intake is possible if the abscess cavity shrinks. In addition, as in this case, if the abscess is located in the upper part of the stomach, it is unlikely that food will move against gravity and pass through a narrow tube into the abscess cavity; therefore, we believe it is likely possible for patients to start eating earlier. Although there are no clear guidelines for when to remove the drain, a fistula generally forms in approximately 1 month, and it is possible to remove the drain at that time.

Depending on the severity and cause of the subdiaphragmatic abscess, the treatment method needs to be examined for each case. The number of elderly patients is expected to increase, and EUS-guided drainage is considered a useful method because it is less invasive. However, few reports have evaluated this method, and it is necessary to study the approach in future cases. As a drainage method for intraabdominal abscess, this approach is definitely considered a minimally invasive and convenient method that should be considered.

## Conclusions

We experienced a case of subdiaphragmatic abscess associated with a perforated duodenal ulcer that was cured by EUS-guided drainage. Drainage is the basic treatment for subdiaphragmatic abscesses, but percutaneous drainage is often difficult because of the anatomy. EUS-guided transgastric drainage is minimally invasive if the lesion is adjacent to the stomach, and this method is considered safe and effective.

## Data Availability

Not applicable.

## Abbreviations

EUS: Endoscopic ultrasound
CT: Computed tomography
